# Driver Countries in Global Banking Network

**DOI:** 10.3390/e22080810

**Published:** 2020-07-23

**Authors:** Farzaneh Atyabi, Olha Buchel, Leila Hedayatifar

**Affiliations:** 1 Department of Physics, North Tehran Branch, Islamic Azad University, Tehran 1477893855, Iran; farzaneh.atyabi@gmail.com; 2 New England Complex Systems Institute, 277 Broadway, Cambridge, MA 02139, USA; olha@necsi.edu

**Keywords:** cross-border bank lending network, driver countries, dimension reduction, principal component analysis

## Abstract

We analyze the network of cross-border bank lending connections among countries from 1977 to 2018. The network includes core countries that lend money and peripheral countries that borrow money from core countries. In nowadays highly connected banking network, financial crisis that start from a country can spread to other countries very fast and cause global affects. We use principal component analysis (PCA) to find the influential lending (core) countries in this network over the years and clusters of borrowing (peripheral) countries related to these impactful core countries. We find three clusters of peripheral countries, with some constant and some changing members over time. This can be a sign of changes in the financial or political interactions among countries. The changes in the role of core countries and how these roles get affected by the important financial crisis in the past decades is investigated. Among 31 of core countries, 7 countries have a partially or constantly important role in the network including France, United Kingdom, United States, Japan, Germany, Chinese Taipei and Switzerland.

## 1. Introduction

The global financial-economic system with various economic channels between countries is a highly complex system with intricate inter-dependencies. Cross-border bank lending activities have experienced remarkable growth over the past three decades [1,2,3]. As a key component, internationally connected banking system plays an important role in the global financial architecture [4]. While connecting to the global banking system produces more financial opportunities for countries, the number of connections and structure of the network can contribute to the transmission of financial shocks and impose significant constraints on the dynamics of the crisis spreading [5,6,7,8]. Position of countries in this network, quantity and quality of their connections to the rest of the sectors of the network affect crisis enduring of the countries [9,10,11]. Understanding the topology of the network of cross-border financial flows and defining its influential components over time provides a better assessment of financial stability and systemic risk [12]. So studying the amount of influence of lending countries globally and the group of borrower countries that interact mostly with some specific lenders is crucial.

Advanced economies play the main roles in global banking network (GBN) with a large number of financial flows circulating among themselves and towards emerging and developing economies [13]. Countries with an advanced economy are the ’core’ of the global banking network [14]. The market has made a key financial source for the emerging and developing economies as well [14]. Countries with an emerging and developing economy that borrowing money from the core countries are called ‘periphery’. Due to the coupled structure of the GBN, it is important to study its behavior and structure in aggregate. In this regard, the features of the GBN and dynamics of the topology of interlinkages have been explored using different metrics of network science including measures of country centralities (degree and strength), network density and clustering with useful crisis-signal information [15,16,17]. The results show that not only the global connectivity between countries but also the clustering coefficient of countries increased exactly before the financial crisis in 2007 [10].

One of the powerful methods to analyze complex systems with many interacting features is the principal component analysis (PCA). It has been used for analyzing the complexity of large data sets in many different disciplines and found a wide range of applications in financial systems. In the cross-border banking networks, PCA has been applied to the transformation of the financial indicators or measurements of countries to detect early-warning signals of crisis [10,18]. Also, PCA has been applied to identify the impactful trading factors of stocks [19], to reduce multicollinearity of key variables in currency exchange rate in some asian countries against the US Dollar [20], and in financial analysis OF real estate companies [21]. In Ref. [22], using PCA, researchers studied principal components that have important implications on the portfolio management and the systemic risk of the stock market and found leading indicators in the financial crisis. In Ref. [23], PCA results suggested how to optimize portfolio investments and find the best way to get financial risk controls and secure high returns.

Since changes in cross-border bank lending strategies, constructed by core countries, play a very important role in shaping economic fluctuations and lead to spillovers of financial conditions to other advanced and emerging market economies [24,25,26], in this paper we focus on this influential financial instrument. We have analyzed the structural properties and time-evolution of the global cross-border bank lending network for 223 countries over 1977–2018. We used PCA to study how groups of borrower countries (peripheral countries) contribute to the specific lending countries (core countries) over the years. Moreover, we show the changes in the role of core countries and how these roles get affected by the important financial crisis in the past decades.

## 2. Materials and Methods

### 2.1. Data

We construct the global banking network using data on cross-border bank lending system from the Bank for International Settlements (BIS) and BIS locational banking statistics [27]. Since locational statistics are collected on the basis of residence of reporting banks, they are suited for analyzing geographical financial linkages and temporal driver countries. The data set becomes larger as lending information of countries is collected after their financial sector starts growing and get invited to report their records to BIS [10]. The data represent all reporting banks/institutions (domestic, foreign, consortium and unclassified) for all currencies and show the annual activities of lending countries that reach 31 in 2018. The total number of borrowing countries increases from 143 in 1977 to 223 in 2018. But this incompleteness does not make any problem as we have the required information about the influential countries. The 31 countries that have lent money to other countries are considered as the core countries. For the non-reporting countries, we only have access to their borrowing information from the core countries. As core countries borrow money from each other as well, we have them in the list of borrowing countries. Borrowing countries are called peripheral countries.

For each year, we built a scaled matrix of connections between core and peripheral countries, named X. Rows and columns of the matrix represent peripheral and core countries respectively and connections represent a given loan by a core country to a peripheral country. We used PCA to analyze the role of countries in the yearly financial transactions. Matrices are scaled separately each year by keeping the range of interactions (given loan from a core country to a peripheral country) in [0,1], where 0 refers to no financial interaction between the two countries and the largest amount of the loan given is scaled to be 1. The scaling is done to keep the analysis consistent over the years as the data show a large growth in the magnitude of cross-border financial flows. So in the matrix of each year, the largest given loan in the year has value 1 and loans less than the maximum one are <1. Cells that countries in them don’t have any lending activity have 0 value.

### 2.2. Principal Component Analysis

Principal component analysis (PCA) is a dimension reduction method for finding patterns in data of high dimension, proposed by Karl Pearson [28] and developed by Harold Hotelling [29]. PCA is applied to extract information from a data table representing a set of observations described by several possibly inter-correlated variables and express this information as a set of few orthogonal uncorrelated variables called principal components. By projecting data onto lower dimensions, while retaining trends and patterns, PCA seeks to interpret, explore and visualize the data in a more meaningful form and observe outliers, clusters and time-based patterns inherited from multivariate data which are too difficult to identify without performing the PCA [29,30,31,32,33]. Some other applications for multivariate data analysis are in medicine [34,35,36,37,38,39], agriculture [40], geology [41], psychology and sociology [42], image processing [43,44], social behaviors [45] and face recognition [46,47].

We now briefly review the PCA algorithm. Consider a data set of *n* observations (samples) with *m* features (variables) that can be assembled into n×m matrix, $\tilde{X}=\left({\tilde{X}}^{1},{\tilde{X}}^{2},\ldots ,{\tilde{X}}^{m}\right)$. Where ${\tilde{X}}^{i}$ denotes the *i*th column of $\tilde{X}$. Subtract the mean from each of the columns to get the matrix $X=(X^{1},X^{2},\ldots ,X^{m})$ with zero mean, where $X^{i}={\tilde{X}}^{i}-\bar{X^{i}}$ with $\bar{X^{i}}=1/n\sum_{j}\left(X_{ji}\right)$. PCs are eigenvectors of the covariance matrix $X^{T}X$, named $P^{i}\left(i=1,\ldots ,m\right)$, arranged from larger to smaller corresponding eigenvalues. The set of PCs represents the amount of variation in the data. By choosing *d* eigenvectors with the largest eigenvalues, a m×d matrix $P=(P^{1},P^{2},\ldots ,P^{d})$ is built where the *d* more significant PCs are the columns. The PCs are a new basis for representing the original data set. The matrix T=XP re-expresses our data in new coordinates which gives us PC scores. An important point with PCA is orthonormality of *P* which results in preserving correlation in *X* that is desirable to us. Indeed it can be shown that by choosing *P* as mentioned, linearly transforming the original data is accompanied by minimizing the covariance between new variables and maximizing the variance which means diagonalizing the covariance matrix of the transformed data.

We perform PCA on our data to identify patterns in the data by highlighting the similarities and differences in the role of countries in the global bank lending network. In our dataset, *n* peripheral countries are our samples which the number might be different each year, n<224. Each of the countries is defined by 31 different variables (related to 31 core countries that lending money), so *X* is a n∗31 matrix for each year. Each cell $X_{ji}$ indicates the given loan (sum of all currencies) by a core country *i* to a peripheral country *j*. PCA finds a new coordinate system in which every peripheral country (samples) has new variables (PCs), which are linear combinations of old coordinate.

## 3. Results and Discussions

The international bank lending system has experienced significant growth in the number of connections and the magnitude of flows over the past decades. Net cross-border bank lending annually surpasses trillions of dollars in recent years. It has been shown that while a higher density of connections in the financial network increases shock repercussions in the whole system, it also reduces the risk of contagion by absorbing consequences of shocks and share it with more agents in the financial network [48,49,50]. Reaction to the macroeconomic shocks and system fragility to the risks are highly correlated with the patterns of the connections [51,52,53,54]. Minoiu and Reyes [16] showed that over the past decades, while the system has experienced remarkable growth in interconnections and their strength, notable drops recognized during the financial crisis. These waves are getting stronger representing the stronger impact of the global financial crisis. They defined three global waves in cross-border bank lending. The largest decline happened in the economic crisis during 2007–2008. The other two waves refer to the global economic recession in the early 1980s and Asian crisis during 1997–1998.

To have a better knowledge about the patterns of topological connections in the financial markets and a better prediction of the system’s reactions in different situations, it is worthwhile to know the significant players of the network and clusters of markets that are related to these players. Using the PCA method, we study the influential core countries that inject money into the system and clusters of peripheral countries that receive liquidity from core countries.

**Figure 1:**
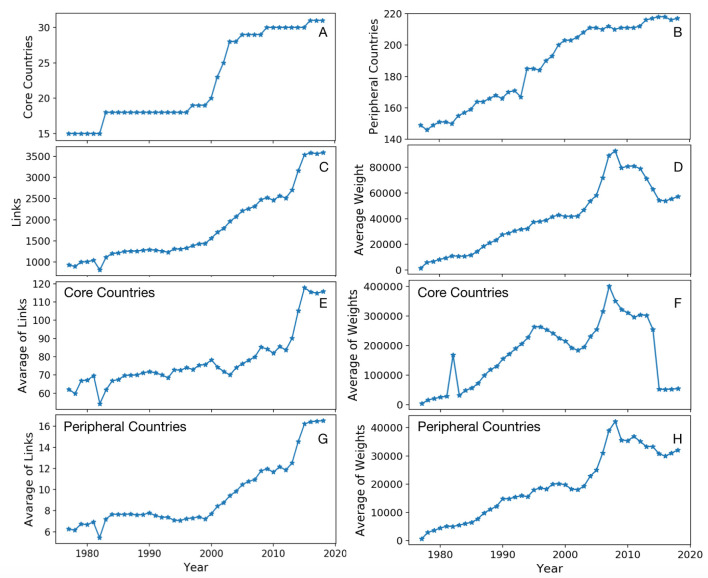
Trends of linkage growth in global bank lending network. (**A**,**B**) show the number of core and peripheral countries over the years. (**C**,**D**) represent the total number of links and average link weights. (**E**–**H**) show the average number of links and weights for core and peripheral countries.

Figure 1 represents trends in links and their weights for core and peripheral countries over the years. While the number of reporting countries shows rapid growth in the early 2000s (Figure 1A), the number of peripheral countries shows almost a linear growth until 2005 and drastically slows down after that (Figure 1B). By having more reported information in 2000, the network shows a linear growth in the total number of links that do not change during the financial crisis in 2007–2008, (Figure 1C). While rapid growth in the number of links in the middle of 2010s can be a sign of the economic prosperity, the average weight of links represents a severe decline, (Figure 1D). Figure 1E,F represent the average number of links and their weights for the core countries. In order to reduce the risks and impacts of the financial crisis, after the crisis in 2008, core countries have decided to make more connections and instead reduce the amount of the loans. According to Figure 1G, the average number of receiving links from core countries to peripheral ones did not change until 2000, and after that, it shows a slight growth. During the financial crisis in 2008 and afterward number of connections does not change, but the average weight of them shows a rapid growth before the crisis and slows down after a peak in 2008.

**Figure 2:**
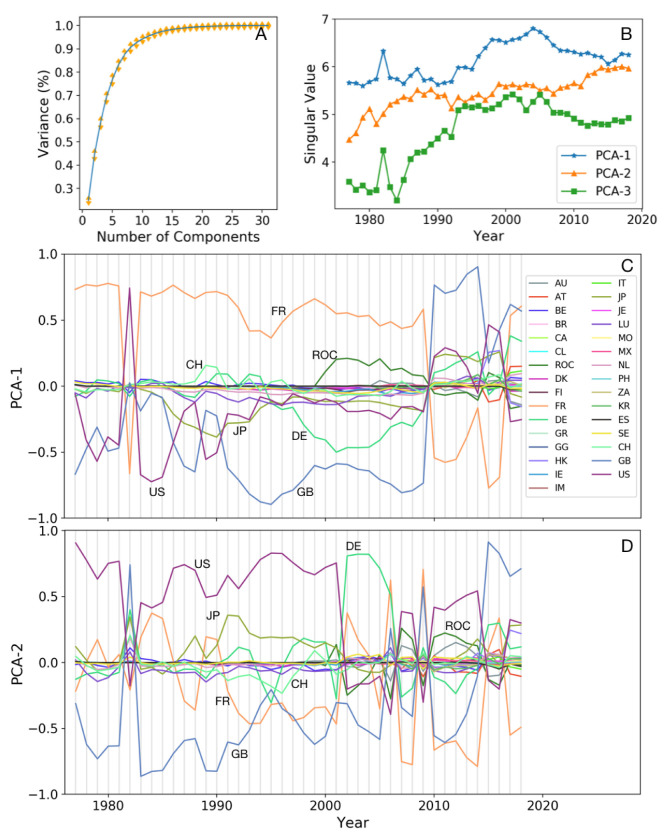
(**A**) The average percentage of variance for all PC components. (**B**) Evolution of singular values for the first three PC components. (**C**,**D**) Role of the core countries in the global bank network over the years 1977–2016 according to PC-1 and 2. The legend on the panel (**C**) defines the countries with their abbreviation and a color. Among all countries, 7 countries have a significant role; France (FR), United Kingdom (GB), United States (US), Japan (JP), Germany (DE), Chinese Taipei (ROC) and Switzerland (CH). See Table 1 for the full name of other countries.

By applying PCA on a matrix of peripheral (223) × Core (31) countries, we are willing to find the influential core countries in the global banking network and clusters of peripheral countries that are connected mostly to some specific core countries. When the system is big and there are many connections that revealing the patterns are not possible with simple procedures like summing or averaging the data, PCA is a method that by reducing the dimension of the data can reveal the hidden patterns of the system. The goal of PCA is reducing features and expressing important information hidden in the system by preserving the essence of the original data. The importance of the PC components depends on their singular value that is descending towards PCs with smaller eigenvalues. However, there is not a magic number of components to consider [55]. In Figure 2a, we calculate the percentage of variance for all the PC components. It shows that in all the years less than 10 components keep around 80% of the total variance of the data and the two first components preserve around 40% of the information. Figure 2b shows the singular values for the first three components in each year. Except for the mid-1990s to mid-2000s, there is a difference of one order of magnitude between the singular value of the third component from the two first components. So in our analysis, we consider the two first components.

Figure 2C,D show the role of the core countries in the network of global banking over the years according to the PC−1 and PC−2. Each country is shown in a different color. The legend in panel (C) shows the countries by their abbreviation name, see Table 1 for the full name of countries. PCA shows that among 31 core countries, 7 countries are major outliers indicating a more influential role in the bank lending network. The uncovered differences come from the higher dimensional data which are projected in the two dimensions and now can be detected more clearly. Some countries play an important role in all the years such as France (FR), United Kingdom (GB), United States (US), and some countries are impactful in a shorter period such as Japan (JP), Germany (DE), Chinese Taipei (ROC) and Switzerland (CH). Dominant positive and negative values for PCs are switching among France (FR), United Kingdom (GB), United States (US) and Germany (DE), reflecting their impact on different sets of peripheral countries. During 1977–1984 and 1986–2009, PCs are almost consistent and in the other years, some fluctuations are observed. According to Figure 2C,D, the Asian crisis during 1997–1998 did not affect the impact of influential core countries. But due to the global economic recession in 1982, the sign of the countries reversed and United Kingdom (GB), that competing with United States (US), lost its impact in the first PCA component. During the global economic crisis in 2007–2008, while PCA-1 just shows reverse in the signs of the countries after the crisis, in PCA-2, fluctuations in the country’s roles start happening few years before the crisis begins. PCA-2 shows that the US lost its impact in early 2000 and Germany (DE) became dominant until 2006.

**Figure 3:**
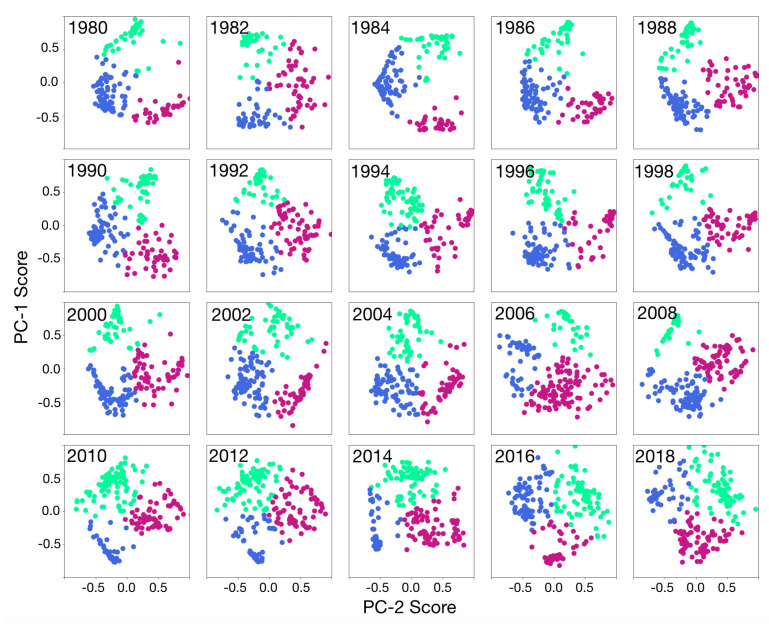
Clustering of the peripheral countries in the first two PC-scores space. Values of PC-1 and PC-2 are according to the first two columns of T, for each year. Each color in the panels shows a cluster.

**Figure 4:**
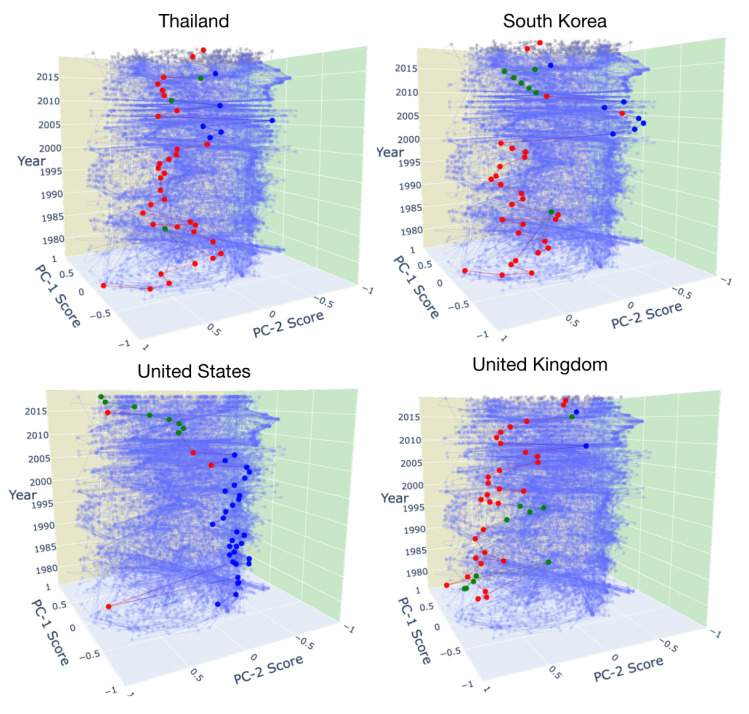
Position of four typical peripheral countries in the PC scores space over the years; Thailand, South Korea, United States, and United Kingdom. Colors represent the cluster a country belongs to it each year.

Any similarity between the peripheral countries will emerge as correlated points being clustered close together in the PC spaces. In Figure 3, peripheral countries are presented in a two-dimensional chart by their first and second principal component (PC) scores over the even years from 1980 to 2018. Colors in each panel represent clusters of countries that are found by applying the K-means algorithm as a clustering technique. Three clusters of the peripheral countries are found by proximity based on their characteristics. While many countries overlapping in the center of the clusters, some of the countries are in the borders of the clusters showing a relation between these countries with effective core countries in the neighboring cluster. To avoid overlapping names, the country names have not been shown, instead one can track each country’s positioning in the clusters over the years in Figure 4. As shown in Figure 3 panels, the position of clusters is almost consistent in the PC score space. The green cluster represents countries that have large positive PC-1 scores and PC-2 scores close to zero. The blue cluster mostly represents countries with negative PC-1 and PC-2 scores, and the red cluster mostly shows countries with close to zero PC-1 score and large positive PC-2 scores. However, a shift in the cluster locations is shown in recent years.

Figure 4 shows the evolution of whole peripheral countries in the PC-1 and PC-2 scores in light blue color. In this figure, we can focus on each country individually. Four typical countries are shown; Thailand, South Korea, United States, and United Kingdom. Colors show the cluster a country belongs to it each year which is consistent with the colors in Figure 3. Most of the countries have not changed their bank relations and stay in the same cluster for many years. The Asian financial crisis was a period (1997–1998) that affected many countries in East and Southeast Asia especially Thailand, South Korea, and Indonesia. Figure 4 shows that both Thailand and South Korea have changed their cluster after the Asian crisis which means changes in their bank relations with some lending countries. The financial crisis in 2007–2008 was the most severe worldwide economic crisis in recent years that started in the United States. While the US is one of the impactful lending countries (see Figure 2), it has also been a large borrower country. According to Figure 2, until 2005, the United States was in the blue cluster, after that and during the financial crisis, it slowly turned to be part of the green cluster. The same happens in Bahrain, Germany, and France. While many countries have changed their banking relations over the years and appeared in new clusters, some of the countries like the United Kingdom have mostly stayed in the same cluster.

## 4. Conclusions

Banking relations can be affected by economic or political events on a global scale. As the world is becoming highly connected over recent decades, financial crises that start from a country or some countries can spread to other countries very soon and have global impacts. So, it is important to know the characteristic behaviors of global networks and have knowledge about their impactful components. In recent years, the structure of the global banking network was analysed using methods like centrality measurements and community detection algorithms. Here, we have developed a method to detect the influential lending countries and clusters of borrowing countries. We have applied principal component analysis (PCA) to the yearly matrices of lending (core) and borrowing (peripheral) countries from 1977–2018. It is shown that among 31 core countries, 7 of them partially or constantly have had an important role including 7 countries have a significant role; France (FR), United Kingdom (GB), United States (US), Japan (JP), Germany (DE), Chinese Taipei (ROC) and Switzerland (CH).

## Figures and Tables

**Table 1:** List of core countries with their abbreviation.

Abbreviation	Country Name	Abbreviation	Country Name
AU	Australia	IT	Italy
AT	Austria	JP	Japan
BE	Belgium	JE	Jersey
BR	Brazil	LU	Luxembourg
CA	Canada	MO	Macao SAR
CL	Chile	MX	Mexico
ROC	Chinese Taipei	NL	Netherlands
DK	Denmark	PH	Philippines
FL	Finland	ZA	South Africa
FR	France	KR	South Korea
DE	Germany	ES	Spain
GR	Greece	SE	Sweden
GG	Guernsey	CH	Switzerland
HK	Hong Kong SAR	GB	United Kingdom
IE	Ireland	US	United States
IM	Ise of Man		
